# Gender difference in the association between TyG index and subclinical atherosclerosis: results from the I-Lan Longitudinal Aging Study

**DOI:** 10.1186/s12933-021-01391-7

**Published:** 2021-10-13

**Authors:** Ya-Wen Lu, Chun-Chin Chang, Ruey-Hsing Chou, Yi-Lin Tsai, Li-Kuo Liu, Liang-Kung Chen, Po-Hsun Huang, Shing-Jong Lin

**Affiliations:** 1 Division of Interventional Cardiology, Cardiovascular Center, Taichung Veterans General Hospital, 1650 Taiwan Boulevard Sect. 4, Taichung, Taiwan; 2 Division of Cardiology, Department of Medicine, Taipei Veterans General Hospital, Taipei, Taiwan; 3 Present Address: Institute of Clinical Medicine, Cardiovascular Research Center, National Yang Ming Chiao Tung University, Taipei, Taiwan; 4 Department of Critical Care Medicine, Taipei Veterans General Hospital, Taipei, Taiwan; 5 Center for Geriatrics and Gerontology, Taipei Veterans General Hospital, Taipei, Taiwan; 6 Aging and Health Research Center, National Yang Ming Chiao Tung University, Taipei, Taiwan; 7 Institute of Public Health, National Yang Ming Chiao Tung University, Taipei, Taiwan; 8 Taipei Municipal Gan-Dau Hospital, Taipei, Taiwan; 9 Taipei Heart Institute, Taipei Medical University, Taipei, Taiwan; 10 Division of Cardiology, Heart Center, Cheng-Hsin General Hospital, Taipei, Taiwan

**Keywords:** TyG index, Insulin resistance, Carotid intima-media thickness

## Abstract

**Background:**

Insulin resistance (IR) is a known risk factor for cardiovascular disease (CVD) in non-diabetic patients through the association of hyperglycemia or associated metabolic factors. The triglyceride glucose (TyG) index, which was defined by incorporating serum glucose and insulin concentrations, was developed as a surrogate marker of insulin resistance. We aimed to investigate the association between the TyG index and the early phase of subclinical atherosclerosis (SA) between the sexes.

**Methods:**

The I-Lan Longitudinal Aging Study (ILAS) enrolled 1457 subjects aged 50–80 years. For each subject, demographic data and the TyG index {ln[fasting triglyceride (mg/dL)  ×  fasting plasma glucose (mg/dL)]/^2^} were obtained. Patients were further stratified according to sex and the 50th percentile of the TyG index (≥  8.55 or  <  8.55). SA was defined as the mean carotid intima-media thickness (cIMT) at the 75th percentile of the entire cohort. Demographic characteristics and the presence of SA were compared between the groups. Logistic regression analysis was performed to assess the relationship between TyG index and SA.

**Results:**

Patients with a higher TyG index (≥  8.55) had a higher body mass index (BMI), hypertension (HTN) and diabetes mellitus (DM). They had higher lipid profiles, including total cholesterol (T-Chol) and low-density lipoprotein (LDL), compared to those with a lower TyG index (<  8.55). Gender disparity was observed in non-diabetic women who had a significantly higher prevalence of SA in the high TyG index group than in the low TyG index group. In multivariate logistic regression analysis, a high TyG index was independently associated with SA in non-diabetic women after adjusting for traditional risk factors [adjusted odds ratio (OR): 1.510, 95% CI 1.010–2.257, *p*  =  0.045] but not in non-diabetic men. The TyG index was not associated with the presence of SA in diabetic patients, irrespective of sex.

**Conclusion:**

A high TyG index was significantly associated with SA and gender disparity in non-diabetic patients. This result may highlight the need for a sex-specific risk management strategy to prevent atherosclerosis.

**Supplementary Information:**

The online version contains supplementary material available at 10.1186/s12933-021-01391-7.

## Introduction

Insulin resistance (IR), a component of metabolic syndrome (MetS), may precede type 2 diabetes mellitus (T2DM) for decades. It is also an independent risk factor for cardiovascular disease (CVD) in patients without T2DM [1–3]. Moreover, IR is responsible for up to 40% of myocardial infarction and is the most important single risk factor for coronary artery disease (CAD) in young adults [4]. To represent IR, the homeostasis model assessment of IR (HOMA-IR) is a validated and frequently used marker by the incorporation of serum glucose and insulin concentrations. However, it has limited application in clinical practice due to the atypical measurement of serum insulin levels [5]. The fasting triglyceride and glucose (TyG) index was first reported in 2008 as a valuable marker of IR, with a close relationship with HOMA-IR [6]. Numerous clinical studies have shown that a higher TyG index is linked to an increased risk of CVD and a greater prevalence of major adverse cardiovascular events in patients with acute coronary syndrome [7, 8].

Carotid intima-media thickness (cIMT) is accepted as an ultrasound marker of early phase subclinical atherosclerosis (SA) and could be a predictive factor of future CVD events [9, 10]. The well-known determinants of thicker cIMT are age, hypertension (HTN), and sex. Diabetic patients exhibit thicker cIMT than those without DM. In addition, individuals with impaired glucose tolerance but without DM also show a thicker CIMT, although to a lesser extent [11]. Females had a lower thickness of cIMT compared with males in various studies, including the Gutenberg-Heart Study in Germany [12], Atherosclerosis Risk in Communities (ARIC) study [13], and the Suita study for Japanese subjects [14].

Sex disparities exist in glucose metabolism. As previous studies have shown, T2DM is a greater risk factor for ischemic heart disease in women than in men. Impaired glucose tolerance is more prevalent in women [15]. However, clinical investigations on the impact of IR on the early phases of SA between men and women remains scarce. The purpose of this study was to investigate whether the TyG index, as a surrogate marker for IR, is associated with the early phase of SA. We also investigated whether sex disparities exist, especially in the non-diabetic stage.

## Methods

### Study population

**Fig. 1:**
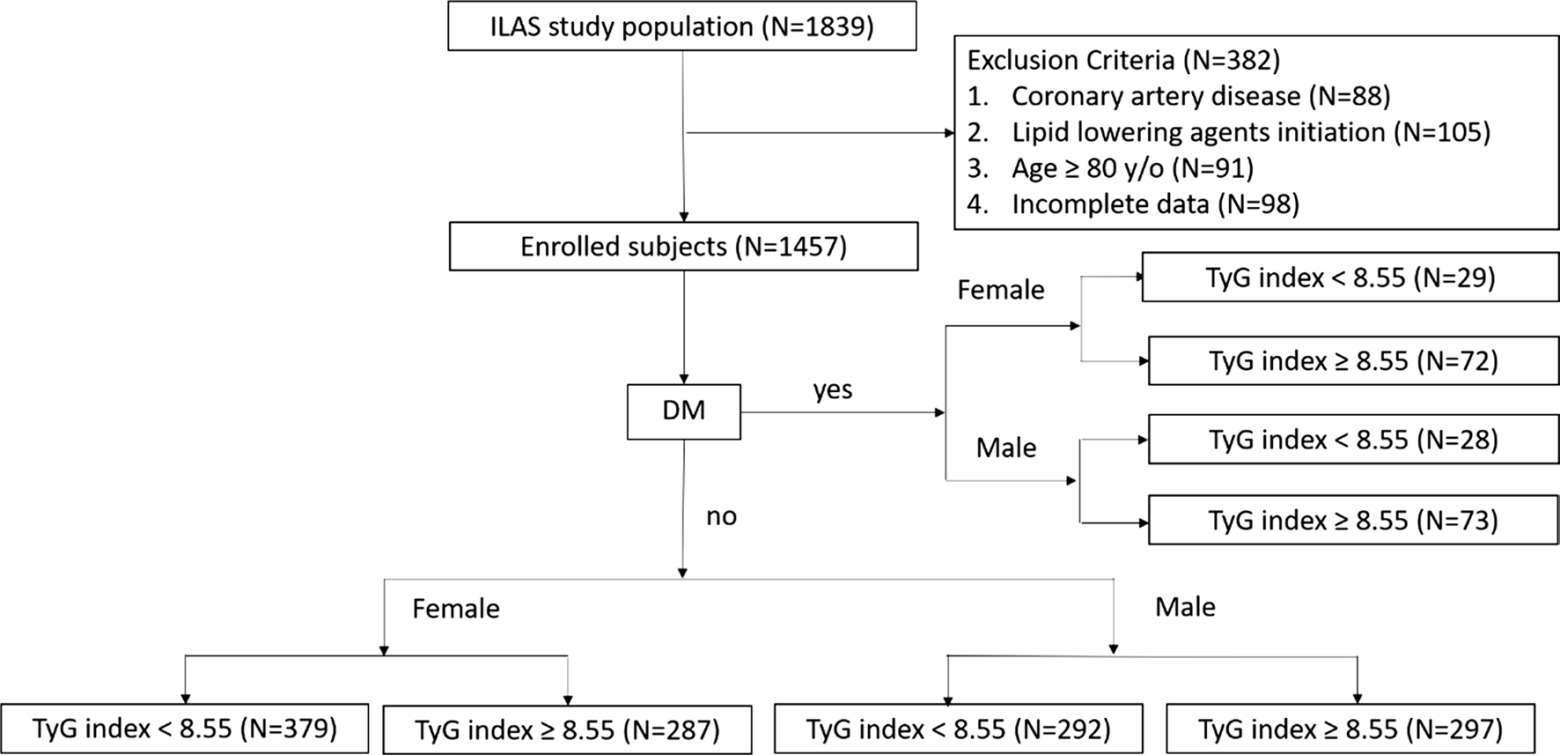
Flow chart for patients enrollment

The present cross-sectional study was conducted with participants from the first wave of the I-Lan Longitudinal Aging Study (ILAS). The ILAS is a research cohort of community-dwelling adults aged  >  50 years who were randomly recruited through household registration records. Inhabitants who met the study inclusion criteria were randomly sampled from the household registration data of the county government. Selected inhabitants were invited to participate via mail or telephone. The inclusion criteria were (1) those having no plans to move out of I-Lan County in the near future and (2) those aged 50 years or older. Details of the ILAS design, participant recruitment, and data collection have been reported previously [16]. The participants were excluded from this study if any of the following conditions were met: (i) they were unable to cooperate or communicate with study investigators, (ii) they declined to grant consent, (iii) they were currently institutionalized, or had any known active disease, such as active cancer, sepsis, heart failure, chronic obstructive pulmonary disease, or functional dependence; (v) they had a life expectancy of less than six months; or (vi) they planned to leave I-Lan county. A total of 1839 community-dwelling older adults were enrolled between August 2011 and August 2013. All participants provided written informed consent after receiving in-person face-to-face interviews with well-trained research nurses. Among them, 382 subjects were excluded, including 88 with CAD, 105 taking lipid-lowering agents, 91 older than 80 years, and 98 with incomplete data (Fig. 1). The study was conducted in accordance with the Declaration of Helsinki and was approved by the Institutional Review Board of the National Yang Ming Chiao Tung University (YM103008).

### Anthropometry, demographic measurements, and laboratory examinations

A research nurse used a questionnaire to collect data regarding demographics, years of education, smoking habits, and other elements of the participants’ medical histories. The height, weight, and resting blood pressure (BP) of each subject were measured. Brachial blood pressure was measured with a mercury sphygmomanometer after the subjects had rested for at least 15 min. Basic medical history elements, including underlying diseases, medication, and smoking, were obtained from personal interviews, and recorded in the medical notes. After fasting for at least 10 h, peripheral blood samples were collected from 7 to 9 AM to determine the concentrations of hemoglobin A1c (HbA1c), fasting blood glucose (FBG), T-Chol, high-density lipoprotein (HDL), LDL, triglyceride (TG), uric acid (UA), and high-sensitivity C-reactive protein (hsCRP) using an automatic analyzer (ADVIA 1800; Siemens, Malvern, PA, USA).

Metabolic syndrome (MetS) was defined according to the criteria proposed by Taiwan’s Ministry of Health and Welfare. More than three of the following risk determinants were included: (i) waist circumference  >  90 cm for men or  >  80 cm for women; (ii) systolic blood pressure  ≥  130 mmHg, diastolic blood pressure  ≥  85 mmHg, or use of antihypertensive agents; (iii) HDL  <  40 mg/dL for men or  <  50 mg/dL for women; TG  ≥  150 mg/dL; (iv) FBG  ≥  100 mg/dL or use of antihyperglycemic agents. Chronic kidney disease (CKD) was defined as an eGFR  <  60, which was calculated using the Chronic Kidney Disease Epidemiology Collaboration equation [17]. Individuals were defined as overweight by a BMI more than 25.0 from the WHO uniform categories of BMI [18]. The TyG index was calculated using the following formula: ln[fasting triglyceride (mg/dL)  ×  fasting plasma glucose (mg/dL)/^2^] [6]. The optimal cut-off value of the TyG index to define insulin resistance has not yet been standardized. Similar to previously published studies [19, 20], we used the median value of the TyG index (8.55) to stratify the study population. A flowchart of patient enrollment and classification is illustrated in Fig. 1.

### Assessment of carotid intima-media thickness (cIMT) and subclinical atherosclerosis (SA)

The cIMT was measured using a high-resolution, broad-width, linear array transducer (LOGIQ 400 PRO; GE, Cleveland, OH, USA) at the level of the common carotid artery. All examinations were performed by the same trained technician, who measured the arteries, including the proximal to distal sections of the bilateral common carotid arteries on longitudinal views [21]. The mean cIMT was defined as the average of the right-and left-side cIMT values. The 75th percentile of cIMT was defined as the upper limit to be considered normal, which was the threshold indicative of increased cardiovascular risk and the early phase of SA [10, 22–24]. In the present study, the 75th percentile of cIMT was 0.75 mm.

### Statistical analysis

Data were expressed as frequencies (percentages) for categorical variables and as means  ±  standard deviations for continuous variables with normal distribution and as median with interquartile range due to the non-normal distribution. The chi-square test was used for comparisons between two groups of categorical variables. The independent t test was employed for continuous variables with normal distribution. The Kruskal–Wallis test was used for non-normally distributed continuous variables. Pearson and Spearman’s tests were used to assess the correlation between the TyG index and cIMT with other variables. Associations between factors and SA were expressed as odds ratios (ORs). Factors that were significant in univariate regression analysis (*p*  <  0.05) were entered into the multivariate regression analysis to evaluate the relationship between the TyG index and SA. Odds ratios with 95% confidence intervals (95% CI) for the risk of cIMT  ≥  0.75 mm were reported. Statistical analyses were performed using SPSS (version 22.0; IBM Corporation, Armonk, New York, USA). Two-tailed *p* values  <  0.05, were regarded as statistically significant.

## Results

A total of 1457 patients (47.4% men; mean age 62.34  ±  8.00 years) without CAD, without lipid-lowering agents and less than 80 years old, were investigated. Compared with the lower TyG index (TyG index  <  8.55) group, patients with a higher TyG index (TyG index  ≥  8.55) were more often male, had a higher waist circumference, and a higher prevalence of being overweight (BMI  ≥  25.0). They exhibited MetS, DM, and HTN. The values of lipid profiles including T-chol, LDL, TG, FBG, and HbA1c were significantly increased in the high TyG index group. There was no difference in age, CKD, or smoking history between the two groups. The mean cIMT was 0.68  ±  0.13 mm and significantly thicker in the higher TyG index group. The percentage of cIMT more than 0.75 mm, as the 75th percentile of the whole analyzed group was significantly higher in the higher TyG index group, as shown in Table 1.

**Table 1:** Baseline characteristics stratified by TyG index of entire population stratified by lower and higher TyG index

	Total cases; n = 1457	TyG index < 8.55; n = 728	TyG index ≥ 8.55; n = 729	*p* value
Age (years)	62.34 ± 8.00	62.31 ± 8.06	62.37 ± 7.95	0.894
Female/male (%)	767/690 (52.6/47.4)	408/320 (56.0/44.0)	359/370 (49.2/50.8)	0.010
BMI	24.81 ± 3.55	23.55 (21.66–25.79)	25.35 (23.42–27.65)	< 0.001
Waist circumference	84.28 ± 9.75	81.5 (75.0–88.0)	86.5 (80.0–93.0)	< 0.001
Smoking (%)	260 (17.8)	117 (16.1)	143 (19.6)	0.087
Underlying disease				
Hypertension (%)	520 (35.7)	212 (29.1)	308 (42.2)	< 0.001
Anti-hypertensive agents (%)	239 (16.4)	93 (12.8)	146 (20.0)	< 0.001
DM (%)	202 (13.9)	57 (7.8)	145 (19.9)	< 0.001
CKD (%)	282 (19.4)	129 (17.8)	153 (21.0)	0.127
Metabolic syndrome (%)	453 (31.1)	90 (12.4)	363 (49.8)	< 0.001
Overweight (%)	633 (43.4)	239 (32.8)	394 (54.0)	< 0.001
LDL ≥ 130 mg/dL (%)	554 (38.0)	239 (32.8)	315 (43.2)	< 0.001
Laboratory data				
Total cholesterol (mg/dl)	197.66 ± 35.30	193 (168–215)	202 (177–223)	< 0.001
HDL (mg/dl)	55.16 ± 14.11	58 (50–69)	49 (42–56)	< 0.001
LDL (mg/dl)	120.87 ± 32.57	116 (96–137)	124 (102–144)	< 0.001
Fasting glucose (mg/dl)	101.49 ± 28.16	92 (87–98)	100 (92–111)	< 0.001
HbA1c (%)	6.01 ± 0.98	5.7 (5.5–6.0)	5.9 (5.6–6.4)	< 0.001
Uric acid	5.84 ± 1.50	5.4 (4.6–6.3)	6.1 (5.1–7.1)	< 0.001
hs-CRP	0.22 ± 0.38	0.069 (0.033–0.190)	0.102 (0.041–0.245)	0.975
Triglyceride (mg/dl)	121.85 ± 78.47	77 (60–90)	143 (123–186)	< 0.001
eGFR (ml/min/1.73 m^2^)	83.33 ± 28.30	82.62 ± 27.24	84.03 ± 29.32	0.341
Triglyceride glucose index	8.57 ± 0.58	8.18 (7.95–8.36)	8.88 (8.70–9.21)	< 0.001
Mean cIMT (mm)	0.68 ± 0.13	0.65 (0.60–0.75)	0.70 (0.60–0.75)	0.003
cIMT ≥ 0.75 mm (%)	417 (28.6)	189 (26.0)	228 (31.3)	0.028

Values are mean  ±  standard deviation or *n* (%)

*BMI* body mass index, *DM* diabetes mellitus, *CKD* chronic kidney disease, *HDL* high density lipoprotein, *LDL* low density lipoprotein, *HbA1c* hemoglobin A1c, *hs-CRP* high sensitivity C-reactive protein, *eGFR* estimated glomerular filtration rate, *cIMT* carotid intima-media thickness

^a^Triglyceride glucose index  =  ln[fasting TG (mg/dL)  ×  fasting plasma glucose (mg/dL)/^2^]

**Fig. 2:**
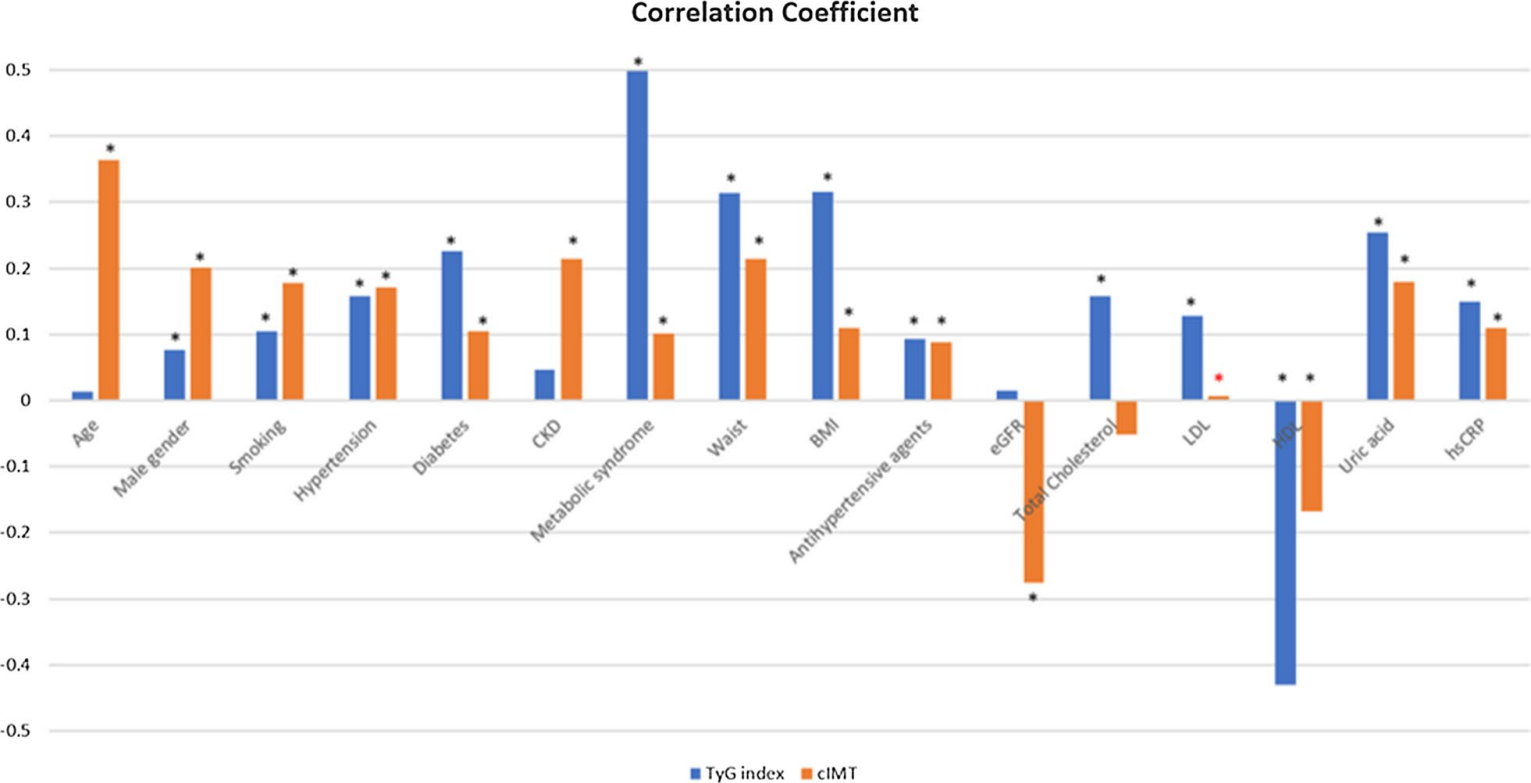
Correlation coefficient of TyG index and cIMT. Black * revealed p value  <  0.001. Red * revealed p value  <  0.05. *BMI* body mass index, *DM* diabetes mellitus, *CKD* chronic kidney disease, *HDL* high density lipoprotein, *LDL* low density lipoprotein, *HbA1c* hemoglobin A1c, *hs-CRP* high sensitivity C-reactive protein, *eGFR* estimated glomerular filtration rate, *cIMT* carotid intima-media thickness

Figure 2 shows the correlation coefficients between the TyG index and cIMT with other variables. Male sex, smoking, HTN, DM, MetS, BMI, antihypertensive drug usage, total cholesterol, uric acid, and hsCRP were positively correlated with both the TyG index and cIMT. Meanwhile, serum HDL levels were negatively correlated with both the TyG index and cIMT. LDL levels were only positively correlated with the TyG index and not with cIMT. In addition, age and CKD were only positively correlated with cIMT, not the TyG index. The values of correlation coefficients of TyG index and cIMT are shown in Additional file 1: Table S1.

We stratified the subjects into non-diabetes and diabetes groups between the sexes. For nondiabetic subjects, as shown in Table 2**,** a greater prevalence of MetS, those that were overweight, had higher levels of T-chol, HbA1c, UA, and a significantly lower HDL were found in the higher TyG index group. This was the case for both males and females (all *p *values  <  0.05). However, a greater prevalence of hypertension (24.3% vs. 37.6%, *p* value  <  0.001), a significantly thicker cIMT (0.64  ±  0.11 mm vs. 0.67  ±  0.11 mm, *p*  =  0.006), and a higher prevalence of SA (17.2% vs. 25.4%, *p*  =  0.012) was observed only in non-diabetic females with a higher TyG index than in subjects with a lower index. For diabetic populations, as Additional file 2: Table S2, both men and women had a higher prevalence of MetS, a greater T-chol level, and a lower HDL. However, only female subjects had significantly higher BMI, LDL, and HbA1c in the higher TyG index population. There was no difference in the prevalence of hypertension, CKD, or individuals who were overweight. There was no cIMT value difference in diabetic females or males stratified by a lower or higher TyG index.

**Table 2:** Baseline characteristics according to lower and higher TyG index in nondiabetic population stratified by gender

	Female			Male		
	Lower TyG index; n = 379	Higher TyG index; n = 287	p value	Lower TyG index; n = 292	Higher TyG index; n = 297	p value
Age (years)	60.94 ± 7.61	61.71 ± 7.64	0.198	63.29 ± 8.21	62.26 ± 8.18	0.126
BMI	23.23 (21.22–25.36)	24.79 (22.92–27.50)	< 0.001	23.78 (21.97–25.85)	25.52 (23.32–27.20)	< 0.001
Waist circumference	78.5 (72–85)	83 (78–90)	< 0.001	84 (79–90)	88 (83–94)	< 0.001
Smoking (%)	9 (2.4)	12 (4.2)	0.262	99 (33.9)	101 (34.0)	1.000
Underlying disease						
Hypertension (%)	92 (24.3)	108 (37.6)	< 0.001	86 (29.5)	102 (34.3)	0.216
Anti-hypertensive agents (%)	42 (11.1)	42 (14.6)	0.195	35 (12.0)	54 (18.2)	0.039
CKD (%)	16 (4.2)	20 (7.0)	0.165	97 (33.4)	94 (31.6)	0.660
Metabolic syndrome (%)	41 (10.8)	141 (49.1)	< 0.001	22 (7.5)	109 (36.7)	< 0.001
Overweight (%)	110 (29.0)	136 (47.4)	< 0.001	102 (34.9)	165 (55.6)	< 0.001
LDL ≥ 130 mg/dL (%)	134 (35.4)	144 (50.2)	< 0.001	96 (32.9)	133 (44.8)	0.003
Laboratory data						
Total cholesterol (mg/dl)	202 (179–222)	211 (191–233)	< 0.001	186 (165–209)	198 (177–218)	< 0.001
HDL (mg/dl)	64 (54–74)	53 (46–60)	< 0.001	53 (46–62)	47 (40–53)	< 0.001
LDL (mg/dl)	118 (98–139)	130 (110–151)	< 0.001	117 (97–137.8)	124 (101.5–143)	0.028
Fasting glucose (mg/dl)	91 (86–96)	97 (91–105)	< 0.001	92 (88–98)	96 (90.5–104)	< 0.001
HbA1c (%)	5.7 (5.5–5.9)	5.9 (5.6–6.2)	< 0.001	5.7 (5.4–5.9)	5.7 (5.5–6.0)	< 0.001
Uric acid	4.9 (4.2–5.6)	5.4 (4.6–6.3)	< 0.001	6.1 (5.3–6.9)	6.7 (5.9–7.7)	< 0.001
hs-CRP	0.067 (0.031–0.179)	0.101 (0.03–0.240)	0.001	0.24 ± 0.48	0.21 ± 0.36	0.361
Triglyceride (mg/dl)	78 (60–91)	141 (124–176)	< 0.001	76 (62–90)	150 (124.5–204.5)	< 0.001
eGFR (ml/min/1.73m^2^)	90.98 (72.30–108.23)	100.30 (79.22–114.24)	0.010	67.01 ± 18.54	70.13 ± 20.26	0.052
Triglyceride glucose index	8.17 (7.90–8.35)	8.83 (8.67–9.07)	< 0.001	8.18 (7.96–8.35)	8.88 (8.71–9.21)	< 0.001
Mean cIMT (mm)	0.64 ± 0.11	0.67 ± 0.11	0.006	0.71 ± 0.16	0.71 ± 0.14	0.698
cIMT ≥ 0.75 mm	65 (17.2)	73 (25.4)	0.012	105 (36.0)	99 (33.3)	0.545

Values are mean  ±  standard deviation or *n* (%)

*BMI* body mass index, *DM* diabetes mellitus, *CKD* chronic kidney disease, *HDL* high density lipoprotein, *LDL* low density lipoprotein, *HbA1c* hemoglobin A1c, *hs-CRP* high sensitivity C-reactive protein, *eGFR* estimated glomerular filtration rate, *cIMT* carotid intima-media thickness

^a^Triglyceride glucose index  =  ln[fasting TG (mg/dL)  ×  fasting plasma glucose (mg/dL)/^2^]

**Fig. 3:**
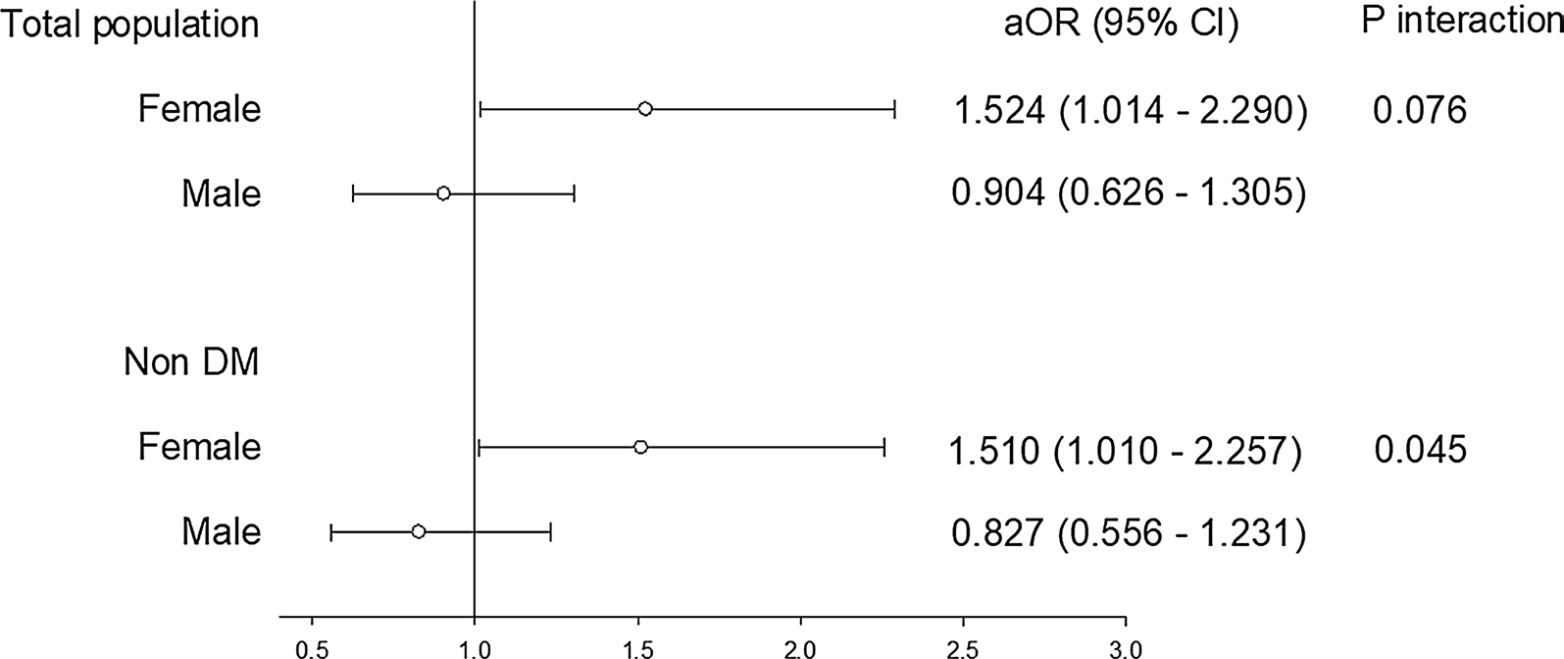
Gender disparity in whole population and non-DM group. *aOR* adjusted odds ratio, variables adjusted: age, BMI, HTN, CKD, UA, hsCRP

Sex disparities were observed in non-diabetic patients (Table 3). Multivariate logistic regression analysis for non-diabetic women showed that a higher TyG index [adjusted odds ratio (aOR): 1.510, 95% CI 1.010–2.257], age (aOR: 1.099, 95% CI 1.070–1.128) and HTN (aOR: 1.689, 95% CI 1.115–2.559) were independently associated with SA (defined as cIMT  ≥  0.75 mm) after adjusting for confounding factors. For nondiabetic men, the independent factors related to SA (defined as cIMT  ≥  0.75 mm) were age (aOR, 1.094; 95% CI 1.067–1.122) and BMI (aOR: 1.118, 95% CI 1.051–1.190). A higher TyG index was significantly associated with cIMT  ≥  0.75 mm in non-diabetic women, however not in non-diabetic men. The *p* for the interaction between the TyG index and gender  =  0.045 (Fig. 3).

**Table 3:** Univariate and multivariate logistic regression analysis of factors associated with the incidence of cIMT  ≥  0.75 mm (75th percentile) in non-DM patients (n  =  1255)

Variable	Univariate analysis			Multivariate analysis		
	OR	95% CI	*p* value	OR	95% CI	*p* value
Female (n = 666)						
Higher TyG index	1.648	1.131–2.402	0.009	1.510	1.010–2.257	0.045
Age	1.106	1.078–1.135	< 0.001	1.099	1.070–1.128	< 0.001
BMI	1.055	1.003–1.109	0.038	1.000	0.940–1.063	0.995
Smoking	1.962	0.776–4.959	0.154			
Hypertension	2.397	1.627–3.532	< 0.001	1.689	1.115–2.559	0.013
CKD	2.597	1.292–5.520	0.007	1.061	0.481–2.338	0.884
Anti-hypertensive agents	1.531	0.909–2.578	0.109			
Total cholesterol	1.004	0.998–1.009	0.173			
HDL	0.990	0.977–1.004	0.167			
LDL	1.004	0.999–1.010	0.138			
UA	1.293	1.115–1.499	0.001	1.117	0.947–1.317	0.189
hs-CRP	1.277	0.751–2.172	0.367			
Male (n = 589)						
Higher TyG index	0.890	0.634–1.251	0.503			
Age	1.086	1.062–1.111	< 0.001	1.094	1.067–1.122	< 0.001
BMI	1.062	1.007–1.119	0.026	1.118	1.051–1.190	< 0.001
Smoking	0.958	0.669–1.373	0.816			
Hypertension	2.022	1.413–2.894	< 0.001	1.319	0.867–2.006	0.196
CKD	1.654	1.157–2.365	0.006	0.991	0.601–1.635	0.972
Anti-hypertensive agents	1.200	0.753–1.912	0.443			
Total cholesterol	0.999	0.994–1.004	0.787			
HDL	0.992	0.978–1.005	0.229			
LDL	1.002	0.997–1.008	0.373			
UA	1.123	0.996–1.265	0.058	1.002	0.890–1.173	0.763
hs-CRP	1.725	1.116–2.668	0.014	1.471	0.945–2.289	0.087

*CKD* chronic kidney disease; *LDL* low-density lipoprotein; *HDL* high-density lipoprotein; *UA* uric acid; *hsCRP* high-sensitivity C-reactive protein

## Discussion

Our results showed that a higher TyG index (TyG index  ≥  8.55) was significantly associated with SA (cIMT  ≥  0.75 mm, as the 75th percentile of the study population) compared with a lower TyG index (TyG index  <  8.55) in women. In addition, the influence of a higher TyG index and SA was only observed in nondiabetic women, however not in nondiabetic men.

### Current evidence of insulin resistance, TyG index, and cIMT

IR is linked to atherosclerosis through metabolic abnormalities, such as hyperglycemia, dyslipidemia, and hyperinsulinemia [25]. HOMA-IR as a surrogate marker of hepatic insulin resistance has been investigated regarding plaque progression in CAD [26]. The disadvantage of the application of HOMA-IR is the atypical measurement of serum insulin levels. The TyG index, as the product of fasting glucose and TG, was strongly associated with HOMA-IR and insulin-stimulated glucose uptake measured as the steady-state plasma glucose concentration during insulin suppression testing [6, 27].

Previous studies have focused on the role of the TyG index in progressive diseases such as carotid atherosclerosis or CVD. This includes CAD, stroke, or peripheral artery disease [28, 29]. Our study provides evidence of the TyG index and the link to SA in a relatively healthy population of middle to old age. We separated the population into women and men due to the excess risk of vascular complications conferred by DM in women [30, 31]. The present study is the first to indicate that gender disparities in the association with SA exist in non-diabetic individuals when the TyG index is increased.

### Sex difference in CHD risk and insulin resistance

Our study showed that the TyG index correlated with metabolic parameters, including waist circumference, HDL, and blood pressure, is in agreement with previous studies. Insulin resistance (or metabolic) syndrome and further cardiovascular complications are believed to be related to adipokines. These include tumor necrosis factor-alpha (TNF-α) and dysregulation. These are involved in the decreased production of nitric oxide in vascular endothelial cells, thus promoting atherosclerosis [32]. The TyG index has been associated with incident hypertension, possibly through insulin resistance-related hyperinsulinemia. This increases sympathetic nervous system activity or activation of the renin–angiotensin–aldosterone system [33, 34].

Women experience greater changes in the rates of BMI and deterioration of lipid profiles compared to men, even when cardiovascular risk factors (CVRF) are similar [35]. The difference between men and women in the preferred location of fat storage could play a role in the development of IR and diabetes [36–38]. Unlike men with more fat stores in the abdominal region with a substantially higher amount of visceral and ectopic fat [39, 40], women are more likely to store fat subcutaneously [41]. As visceral fat is strongly associated with insulin resistance, women may need to gain more weight and experience more significant deterioration of related metabolic risk factors than men to reach the same level of visceral fat storage [42]. This means that sex differences in metabolic risk factors already occur during the transition from normoglycemia to elevated glucose levels and diabetes [31].

In the present study, a higher TyG index in non-diabetic women was significantly associated with SA compared with non-diabetic men. There was no sex disparity in the diabetic group. Enrolled subjects were older than 50 years of age; most women were in late perimenopause or menopause. Endogenous estrogen may play a role in higher insulin sensitivity in females in a rodent model [43]. In large clinical studies, menopausal hormone therapy for postmenopausal women may improve insulin sensitivity through estrogen receptors in the liver, muscle, and adipose tissue [44, 45]. The protective effect of estrogen is eliminated when females have the following risk factors: high ratios of total/high-density lipoprotein cholesterol level ratios, left ventricular hypertrophy, or diabetes [46, 47]. Moreover, not only in DM patients, but also among non-diabetic subjects, women had greater CVD events and death than men. They also had greater effects from a higher TG and blood pressure [15, 47]. In the present study, the greater prevalence of HTN observed in non-diabetic women was consistent with previous studies. The relationship between TyG index and cIMT in nondiabetic women requires further investigation.

### Study limitations

The limitations of our study are as follows: first, due to the retrospective, observational study design, the causal relationship between higher TyG index and SA could not be fully assessed. Second, certain confounding factors, such as duration of diabetes, categories of anti-diabetic agents, or insulin use were not considered in the present study. Third, the cross-sectional cohort study lacked long-term outcome data such as longitudinal studies. Thus, we could not investigate the impact of the TyG index on the risk of new-onset CVD. Fourth, the optimal cut-off value of the TyG index to define insulin resistance has not yet been standardized and may vary between study populations. Lastly, the exploration of the mechanism linking the TyG index and atherosclerosis is beyond the scope of this study.

## Conclusions

In this cross-sectional study, a high TyG index was significantly associated with SA with disparities by sex in non-diabetic patients after adjusting for the traditional risk factors of atherosclerosis. Our findings suggest a sex-specific risk management strategy for preventing atherosclerosis.

## Supplementary Information

**Additional file 1: Table S1.** Correlation coefficients of TyG index and the carotid intima-media thickness with other cardiovascular risk factors in whole population.

**Additional file 2: Table S2.** Baseline characteristics according to lower and higher TyG index in diabetic population stratified by gender.

## Data Availability

The datasets used and analyzed during the current study are available from the corresponding author upon request.

## Abbreviations

aOR: Adjusted odds ratio
BMI: Body mass index
CAD: Coronary artery disease
cIMT: Carotid intima-media thickness
CVD: Cardiovascular disease
CKD: Chronic kidney disease
DM: Diabetes mellitus
eGFR: Estimated glomerular filtration rate
FBG: Fasting blood glucose
HTN: Hypertension
HOMA-IR: Homeostasis model assessment of IR
HDL: High-density lipoprotein
hsCRP: High-sensitivity C-reactive protein
HbA1c: Hemogloblin A1
ILAS: I-Lan Longitudinal Aging Study
IR: Insulin resistance
LDL: Low-density lipoprotein
Mets: Metabolic syndrome
SA: Subclinical atherosclerosis
TyG: Triglyceride glucose
T-Chol: Total cholesterol
T2DM: Type 2 diabetes mellitus
TG: Triglyceride
