# Supplementary material for: Gender difference in the association between TyG index and subclinical atherosclerosis: results from the I-Lan Longitudinal Aging Study
Source: Cardiovasc Diabetol. 2021 Oct 13;20:206. doi: 10.1186/s12933-021-01391-7 (PMC8515653; doi:10.1186/s12933-021-01391-7)
Supplement: Supplementary file 1 — Additional file 1: Table S1. Correlation coefficients of TyG index and the carotid intima-media thickness with other cardiovascular risk factors in whole population. [file 12933_2021_1391_MOESM1_ESM.docx]

**Table s1.** Correlation coefficients of TyG index and the carotid intima-media thickness with other cardiovascular risk factors in whole population

|  | **TyG index** | **cIMT** |
| --- | --- | --- |
| **Clinical profiles** |  |  |
| Age | 0.014 | 0.363^**^ |
| Male gender | 0.077^**^ | 0.201^**^ |
| Smoking | 0.105^**^ | 0.178^**^ |
| Hypertension | 0.158^**^ | 0.171^**^ |
| Diabetes | 0.226^**^ | 0.104^**^ |
| CKD | 0.047 | 0.215^**^ |
| Metabolic syndrome | 0.499^**^ | 0.101^**^ |
| Waist | 0.314^**^ | 0.215^**^ |
| BMI | 0.315^**^ | 0.109^**^ |
| Antihypertensive agents | 0.093^**^ | 0.088^**^ |
| **Biochemical & image studies** |  |  |
| eGFR | 0.015 | -0.275^**^ |
| Total Cholesterol | 0.158^**^ | -0.051^*^ |
| LDL | 0.128^**^ | 0.007 |
| HDL | -0.430^**^ | -0.168^**^ |
| Uric acid | 0.254^**^ | 0.180^**^ |
| hsCRP | 0.149^**^ | 0.109^**^ |
| cIMT | 0.094** | -- |
| TyG index | -- | 0.094^**^ |

* Correlation is significant at the 0.05 level (2-tailed)

** Correlation is significant at the 0.01 level (2 tailed)

Abbreviations: BMI= body mass index; eGFR= estimated glomerular filtration rate; CKD= chronic kidney disease; LDL= low-density lipoprotein; HDL= high-density lipoprotein; hsCRP= high-sensitivity C-reactive protein; cIMT= carotid intima-media thickness.
