# Supplementary material for: Gender difference in the association between TyG index and subclinical atherosclerosis: results from the I-Lan Longitudinal Aging Study
Source: Cardiovasc Diabetol. 2021 Oct 13;20:206. doi: 10.1186/s12933-021-01391-7 (PMC8515653; doi:10.1186/s12933-021-01391-7)
Supplement: Supplementary file 2 — Additional file 2: Table S2. Baseline characteristics according to lower and higher TyG index in diabetic population stratified by gender. [file 12933_2021_1391_MOESM2_ESM.docx]

**Table S2.** Baseline characteristics according to lower and higher TyG index in diabetic population stratified by gender.

|  | **Female** | | | **Male** | | |
| --- | --- | --- | --- | --- | --- | --- |
|  | **Lower TyG index**  **n=29** | **Higher TyG index**  **n=72** | **p value** | **Lower TyG index**  **n=28** | **Higher TyG index**  **n=73** | **p value** |
| Age (years) | 67.53 (62.0-75.75) | 63.16 (56.35-69.18) | 0.013 | 65.19 ± 7.97 | 64.61 ± 8.16 | 0.750 |
| BMI | 25.44 ± 4.14 | 27.29 ± 4.23 | 0.048 | 24.91 ± 3.75 | 26.29 ± 2.97 | 0.056 |
| Waist circumference | 84.59 ± 13.19 | 88.27 ± 10.00 | 0.132 | 88.05 ± 8.95 | 91.25 ± 8.25 | 0.091 |
| Smoking (%) | 0 (0) | 1 (1.4) | 1.000 | 9 (32.1) | 29 (39.7) | 0.647 |
| **Underlying disease** |  |  |  |  |  |  |
| Hypertension (%) | 19 (65.5) | 50 (69.4) | 0.814 | 15 (53.6) | 48 (65.8) | 0.359 |
| Anti-Hypertensive agents (%) | 10 (34.5) | 21 (29.2) | 0.638 | 6 (21.4) | 29 (39.7) | 0.104 |
| CKD (%) | 6 (20.7) | 8 (11.1) | 0.218 | 10 (35.7) | 31 (42.5) | 0.652 |
| Metabolic syndrome (%) | 16 (55.2) | 58 (80.6) | 0.013 | 11 (39.3) | 55 (75.3) | 0.001 |
| Overweight (%) | 16 (55.2) | 47 (65.3) | 0.371 | 11 (39.3) | 46 (63.0) | 0.043 |
| LDL ≥ 130 mg/dL (%) | 3 (10.3) | 22 (30.6) | 0.042 | 6 (21.4) | 16 (21.9) | 1.000 |
| **Laboratory data** |  |  |  |  |  |  |
| Total Cholesterol (mg/dl) | 172.3 (159.0-181.5) | 190.7 (164.3-214.3) | 0.008 | 163.82 ± 35.26 | 180.81 ± 37.60 | 0.041 |
| HDL (mg/dl) | 58.1 (50.5-69.0) | 49.9 (44.0-57.0) | 0.001 | 52.4 (46.3-56.5) | 45.2 (39.5-49.5) | <0.001 |
| LDL (mg/dl) | 98.4 (78.5-106.0) | 117.0 (91.0-136.8) | 0.006 | 101.29 ± 42.42 | 109.15 ± 33.32 | 0.329 |
| Fasting glucose (mg/dl) | 103.35 (90.0-115.0) | 158.85 (118.0-181.5) | <0.001 | 114.8 (94.0-136.0) | 144.6 (118.5-160.0) | 0.002 |
| HbA1c (%) | 6.63 (6.05-7.10) | 7.85 (6.60-8.88) | <0.001 | 7.11 ± 1.24 | 7.56 ± 1.66 | 0.197 |
| Uric acid | 5.67 ± 1.20 | 5.63 ± 1.53 | 0.916 | 6.04 ± 1.33 | 6.35 ± 1.73 | 0.407 |
| hs-CRP | 0.209 ± 0.416 | 0.259 ± 0.273 | 0.512 | 0.268 ± 0.379 | 0.218 ± 0.382 | 0.600 |
| Triglyceride (mg/dl) | 78.3 (65.0-98.0) | 156.3 (104.3-177.0) | <0.001 | 64.7 (50.5-76.5) | 171.7 (108.0-167.0) | <0.001 |
| eGFR (ml/min/1.73m^2^) | 91.70 ± 38.33 | 97.64 ± 31.40 | 0.422 | 70.89 ± 28.46 | 67.12 ± 23.38 | 0.497 |
| Triglyceride glucose index | 8.24 (8.15-8.45) | 9.27 (8.83-9.46) | <0.001 | 8.11(8.12-8.32) | 9.20 (8.74-9.48) | <0.001 |
| Mean cIMT | 0.714 ± 0.155 | 0.685 ± 0.136 | 0.365 | 0.745 ± 0.137 | 0.735 ± 0.127 | 0.737 |
| cIMT ≥ 0.75mm | 7 (24.1) | 19 (26.4) | 1.000 | 12 (42.9) | 37 (50.7) | 0.512 |

Values are mean ± standard deviation or median (25^th^ percentile-75^th^ percentile) or *n* (%). Abbreviations: BMI=body mass index, DM=diabetes mellitus, CKD = chronic kidney disease, HDL=high density lipoprotein, LDL = low density lipoprotein, HbA1c= hemoglobin A1c, hs-CRP= high sensitivity C-reactive protein, eGFR = estimated glomerular filtration rate, cIMT= carotid intima-media thickness.

* Triglyceride glucose index= ln[fasting TG (mg/dL) × fasting plasma glucose (mg/dL)/2]
